# Identification of 5-Fluoro-5-Deoxy-Ribulose as a Shunt Fluorometabolite in *Streptomyces sp*. MA37

**DOI:** 10.3390/biom10071023

**Published:** 2020-07-10

**Authors:** Linrui Wu, Ming Him Tong, Kwaku Kyeremeh, Hai Deng

**Affiliations:** 1 Department of Chemistry, University of Aberdeen, Aberdeen AB24 3UE, UK; linrui.wu3@abdn.ac.uk (L.W.); r01mht13@abdn.ac.uk (M.H.T.); 2 Department of Chemistry, University of Ghana, P.O. Box LG56 Legon-Accra, Ghana; kkyeremeh@ug.edu.gh

**Keywords:** fluorometabolites, 5-FDRul, *Streptomyces* sp. MA37, genetic manipulation, chemo-enzymatic synthesis

## Abstract

A fluorometabolite, 5-fluoro-5-deoxy-D-ribulose (5-FDRul), from the culture broth of the soil bacterium *Streptomyces sp*. MA37, was identified through a combination of genetic manipulation, chemo-enzymatic synthesis and NMR comparison. Although 5-FDRul has been chemically synthesized before, it was not an intermediate or a shunt product in previous studies of fluorometalism in *S. cattleya*. Our study of MA37 demonstrates that 5-FDRul is a naturally occurring fluorometabolite, rendering it a new addition to this rare collection of natural products. The genetic inactivation of key biosynthetic genes involved in the fluorometabolisms in MA37 resulted in the increased accumulation of unidentified fluorometabolites as observed from ^19^F-NMR spectral comparison among the wild type (WT) of MA37 and the mutated variants, providing evidence of the presence of other new biosynthetic enzymes involved in the fluorometabolite pathway in MA37.

## 1. Introduction

**Figure 1:**
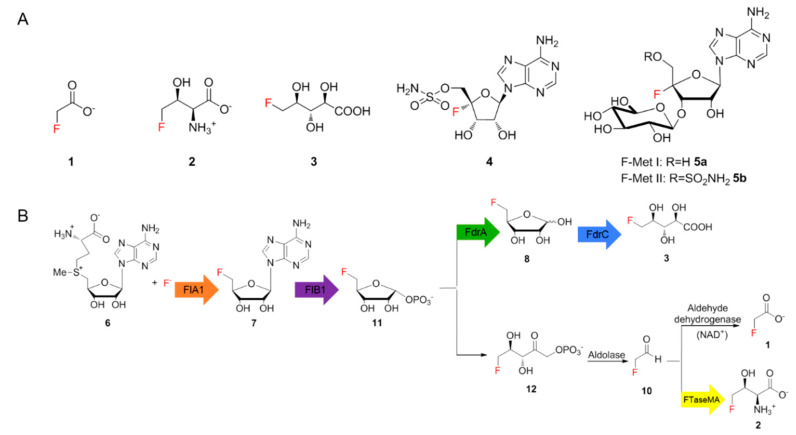
(**A**) Fluorometabolites discovered from bacteria so far. (**B**) The proposed biosynthetic pathways of fluoroacetate 1 and 4-fluorothreonine 2 in *Streptomyces cattleya* and *Streptomyces* sp. MA37, and FHPA 3 in *Streptomyces* sp. MA37.

Fluorinated compounds have remained important in the field of lead compound discovery due to their wide therapeutic and agricultural applications [1]. The unmet need for a fluorine-incorporated lead compound presents an unremitting impetus for the development of selectively efficient fluorination methods. Compared to the vast amount of synthetic fluorinated compounds, naturally occurring fluorinated natural products are extremely rare, most of which are fluorinated fatty acids with different chain lengths discovered in sub-tropical and tropical plants [1]. Fewer than thirty fluorinated natural products have been identified so far, including six structurally different ones of bacterial origin, such as fluoroacetate (FAc) **1**, 4-fluorothreonine (4-FT) **2 [1]**, 5-fluoro-2,3,4-trihydroxypentanoic acid (FHPA) **3** [2], nucleocidin **4** [3] and two new glycosylated nucleocidin derivatives (**5a**, **5b**) (Figure 1A) [4]. Of these, **1** is a toxin that inhibits the citric acid cycle while **2** and **4** were discovered as antimicrobial metabolites. The biological function of **3** in the producing strain remains elusive. The scarcity of fluorinated compounds found in nature, compared to thousands of brominated or iodinated compounds, mainly results from the high electronegativity of fluorine, which precludes the common strategy for halogenation that involves the oxidation of fluoride ions [5]. In addition, the solvation tendency of fluoride ions in aqueous solutions causes fluoride to need extra energy to become a good nucleophile. Such desolvation processes elevate the difficulty of biochemically incorporating fluorine into complex organic molecules [5].

For decades, *Streptomyces cattleya* remained the only genetically tractable strain producing two fluorinated natural products, **1** and **2**. The major breakthrough was the identification of the first fluorination enzyme, fluorinase, which converts *S*-adenosyl-l-methionine (SAM) **6** and fluoride ions to generate 5′-fluoro-5′-deoxy-adenosine (5′-FDA **7**) [6,7]. Subsequent studies resulted in the elucidation of the biosynthetic pathway of **1** and **2** in *S. cattleya* as illustrated in Figure 1B [8]. Recent studies demonstrated that two genes, *fthB* and *fthC*, are highly conserved in the biosynthetic gene clusters (BGCs) that direct the production of **2** [9]. While the genetic inactivation of *fthB* had no perturbation in secreted **2** in the *S. cattleya* variant as observed in ^19^F-NMR analysis, biochemical analysis indicated that FthB, an aminoacyl RNA deacylase, plays an essential role in the detoxification of **2**, by counteracting the misacylation of fluorothreonyl-tRNA which otherwise would be misincorporated into protein in place of L-threonine [9]. While knocking out *fthC* significantly reduced the concentration of **2** in culture broth [9], the intracellular **2** was accumulated in the *S. cattleya* variant, indicating that FthC is a 4-FT exporter. The efforts towards understanding the fluorination enzyme, fluorometabolisms in *S. cattleya* and related biotransformations have offered a new bio-based approach to generating high-value fluorinated chemicals [10].

In the last decade, advanced genome sequencing technologies have enabled the use of genome mining strategies to identify several other potential fluorometabolite-producing strains [2,11]. Recent studies revealed that *Streptomyces* sp. MA37 (MA37), a soil isolate, is a talented natural product producing strain [12,13,14,15]. In particular, MA37 produces **1**, **2** and a series of unidentified fluorinated metabolites as observed in ^19^F-NMR analysis of the supernatant of the culture broth of MA37 [11]. It has been shown that **1** and **2** in MA37 originate from the same biosynthetic pathway as the one in *S. cattleya* [11,16]. A combination of chemical synthesis, bioinformatic analysis and biochemical assays allowed the discovery of **3** as a new fluorometabolite and the identification of the second *fdr* biosynthetic gene cluster (BGC) in MA37 [2]. However, the rest of the fluorometabolites observed in MA37 remain to be determined.

Here we describe the identification of a naturally occurring fluorometabolite, 5-fluoro-5-deoxy-D-ribulose (5-FDRul) **9**, from the culture broth of MA37, using a combination of genetic inactivation, chemo-enzymatic synthesis and ^19^F-NMR comparison. Although reported before as chemical probes during the previous fluorometabolism study in *S. cattleya*, this is the first report indicating that **9** is a naturally occurring fluorinated compound. During the course of our studies, we also observed that the production of several fluorinated natural products was upregulated among several MA37 fluorometabolite biosynthesis related gene inactivation variants as observed in ^19^F-NMR analysis, suggesting that the inactivation of genes responsible for downstream fluorometabolite biosynthesis may influence metabolic reflux and direct accumulated biosynthetic intermediates to other previously unnoticed pathways in MA37.

## 2. Experimental Section

### 2.1. Fermentation Conditions

*E*. *coli* strains were grown in Luria-Bertani (LB) broth (1% tryptone, 0.5% yeast extract, 0.5% NaCl) or LB agar (1.5% agar) at 37 °C, supplemented with the corresponding antibiotics. *E*. *coli* DH10B was used as the routine cloning strain for DNA manipulations. *E*. *coli* ET12567 (pUZ8002), a DNA methylation deficiency stain, served as conjugal donor. MA37 was grown in ISP2 medium and supplemented with apramycin (20 μM) if needed. For mycelia generation, MA37 was inoculated from an ISP2 agar palate to YEME liquid medium (50 mL) and harvested after 2 d shake incubation (180 rpm, 28 °C). For fluorometabolite production, MA37 wild type and the confirmed in-frame deletion variants were grown on ISP2 agar medium for 4 d before being inoculated into ISP2 liquid medium supplemented with KF (2.5 mM). The seed culture was shake-incubated for 2 d (180 rpm, 28 °C). Seed culture (1 mL) was then inoculated into the same ISP2 medium with KF (50 mL) and shake-incubated for 10–14 d. The supernatant of the culture was obtained by removing the mycelium with a centrifugation step (4600 rpm, 20 min) and freeze-dried. The crude extract was supplemented with D_2_O and subjected to ^19^F-NMR analysis after 10–14 d fermentation.

### 2.2. Genomic DNA Isolation

The genomic DNA of MA37 in this study was extracted from 2 mL cell culture. Cell pellet was harvested after 3 d culture in ISP2 medium by centrifugation and resuspended in 500 μL SET buffer (100 mM NaCl, 1 mM EDTA, 10 mM Tris-HCl, pH 8.0). The cell suspension was mixed with lysozyme (4 mg/mL, final concentration) and incubated at 37 °C for 30 min. SDS (60 μL, 10% (*w*/*v*)) and NaCl (200 μL, 5 M) were then added to the mixture, followed by further incubation at 60 °C for 30 min. The protein was precipitated with a mixture of phenol, chloroform and isoamylol (500 μL, ratio of 25:24:1) and the resultant mixture was mixed by vortex. The water fraction was obtained after a centrifugation step and transferred to a new Eppendorf tube with isopropanol for DNA precipitation (0.8 volumes). The precipitated DNA was washed with 75% (*v*/*v*) ethanol, followed by a second wash with 100% ethanol. The DNA pellet was dried at room temperature and dissolved in sterile MiliQ water (200 μL).

### 2.3. The Construction of Plasmids Used for Gene Deletion

To generate the deletion vector for in-frame deletion in MA37, two homologous arms were amplified by PCR using MA37 genomic DNA as a template (*fthCMA* left arm forward: *ggc cag tgc caa gct t*GG AAT GAA CCC CCA GGA GAC CCG CG, reverse: *gcg cag gat acc cgg* ACG GTC GGC CGG CAC TTC GAG GGG; right arm forward: CGG GTG ATC CTG CGC AGG GTC GGG GCG G, reverse: *aca tga tta cga att c*TG CCG GAT CCG CAC GGC CAC GGA GC; *fthBMA* left arm forward: *ggc cag tgc caa gct t*CG CGA AGG GCC CGC GTC CGG TAC AC, reverse: *cac gga aat cac cga* GCC CTA CCG CTG CCC ATG GTG TTC G; right arm forward: TCG GTG ATT TCC GTG CTC TCC GTG G, reverse: *aca tga tta cga att c*TC TCG TTC GCG GTC AGA TGG AGG AC; *fdrA* left arm forward: *ggc cag tgc caa gct t*GC AGG GCG GTG GCG CGT CCG ATG CCG CG, reverse: tcc ccg ttc gcg cag GCA TGT CCT CGA TCT TGC GT; right arm forward: CTG CGC GAA CGG GGA AAC CCC TTC, reverse: *gcg cgg ccg cgg atc c*CG CCA CCG GGC TGA TGG CCC TG; *fdrB* left arm forward: *ggc cag tgc caa gct t*TC TGG TGC CCG ACG GTG ACC GCG AC, reverse: *gca atc tca acc acc* GCC TGA AGG AAT GGG AGG CTC CGC C; right arm forward: GGT GGT TGA GAT TGC ACG GCA T, reverse: *gcg cgg ccg cgg atc c*AG CGC GCC CAG TTC GAG AAC CCC). The homologous arms were ligated to the linearized (*Hin*dIII, *Eco*RI for *fthBMA* and *fthCMA* deletion and *Bam*HI, *Hin*dIII for the rest) temperature-sensitive *E*. *coli*–*Streptomyces* shuttle vector pKC1139 via one pot in-fusion cloning (TAKARA) according to the protocol of the manufacturer (Clontech, TaKaRa, Shiga, Japan). The correct deletion construct was screened by PCR and confirmed by DNA sequencing.

### 2.4. The E. coli–Streptomyces Conjugation and Double-Crossover Variant Generation

The confirmed deletion construct was introduced to *E*. *coli* ET12567 for the *E*. *coli*–*Streptomyces* conjugation. As MA37 is bold and produces no spores, mycelia were used for conjugation instead for spores, and were harvested in YEME culture after 2 d shake incubation. The *E. coli* donor strain (2 mL) was harvested and washed with fresh LB medium (3×, 1.5 mL), before being mixed with MA37 mycelia (200 μL) and spread onto an MS agar plate (Mannitol 2%, Soya bean flour 2%, agar 2%, pH 7.5). The plate was cultured overnight (28 °C, 12–16 h) and overlaid with apramycin and nalidixic acid (50 μM, final concentration). The exoconjugates were purified and single-crossover variants were isolated by incubation at 37 °C to promote plasmid loss. Double-crossover mutant was then acquired by multiple steps of subculture onto an antibiotic-free ISP2 agar plate and confirmed by PCR screening and genomic DNA sequencing.

### 2.5. The Synthesis of 5-FDR

The 5-fluoro-5-deoxy-D-ribose (5-FDR) **8** synthesis protocol was taken from the literature [17,18]. D-(-)-ribose **14** (1.5 g, 1 eq) was added to a round-bottom flask (250 mL) with acetone (80 mL) and cooled under an ice bath. Then, 2,2-dimethoxypropane (2.6 g, 2.5 eq) was added into the mixture. Perchloric acid was added dropwise into the mixture for 5 min. The ice bath was removed and the reaction mixture stirred at room temperature overnight. The reaction mixture turned orange the next morning. Methanol (2.02 mL, 5 eq) was added into the mixture and it was stirred for 3 h. The reaction was quenched by the saturated addition of sodium hydrogen carbonate. The suspension was filtered and rotary evaporated to minimum volume (aqueous phase). The aqueous phase was extracted by ethyl acetate (3 × 100 mL, organic phase). The combined organic phase was dried by anhydrous magnesium sulphate, filtered and dried under vacuum. Then, 1-methoxy-2,2-isopropyliden-α/β-D-ribofuranose 15 (1 g, 1 equiv) was oven-dried and placed under an argon atmosphere system. Dry pyridine (3 mL) was added into the system and the mixture was cooled under an ice bath. After 5 min, *p*-toluenesulfonyl chloride (1.5 equiv) was added into the mixture, which was stirred under the ice bath for 2 h. When the reaction completed, the mixture was poured into 5 mL of ice-cold water with vigorous stirring, and the precipitate formed. The precipitate was filtered, washed by ice-cold water (5 × 5 mL) and dried under a freeze drier. Then, 2,3-O-isopropylidene-5-O-(*p*-toluenesulfonyl)-β,D-ribofuranoside 16 (600 mg, 1.68 mmol, 1 equiv) was added into a flame-dried round-bottom flask and connected to the reflux system. The argon cycle was applied to the system. Anhydrous acetonitrile (7 mL) was added, followed by *tetra*-*n*-butylammonium fluoride (TBAF) (2 mL). The system was heated to 80 °C under reflux overnight. The reaction mixture was dried under vacuum the following morning. The dried product was purified by a silica gel column (mobile phase: petroleum ether:ethyl acetate 10:1). Aqueous sulphuric acid (0.2 M) was mixed into the purified product and heated overnight. The reaction mixture was cooled to room temperature the following morning and neutralized by barium carbonate. The suspension was centrifuged and the supernatant was freeze-dried.

### 2.6. The Generation of 5-FDRul

For the preparation of 5-FDRul **9**, the synthesized 5-FDR **8** (1 mM) was incubated with immobilized glucose isomerase (30 mg) in KH_2_PO_4_ buffer (50 mM, pH 6.8) at 37 °C for 6 h. The enzyme was removed from the reaction mixture by centrifuge (13,000× *g*, 10 min). The supernatant was subjected to ^19^F-NMR analysis. 

## 3. Results and Discussion

**Figure 2:**
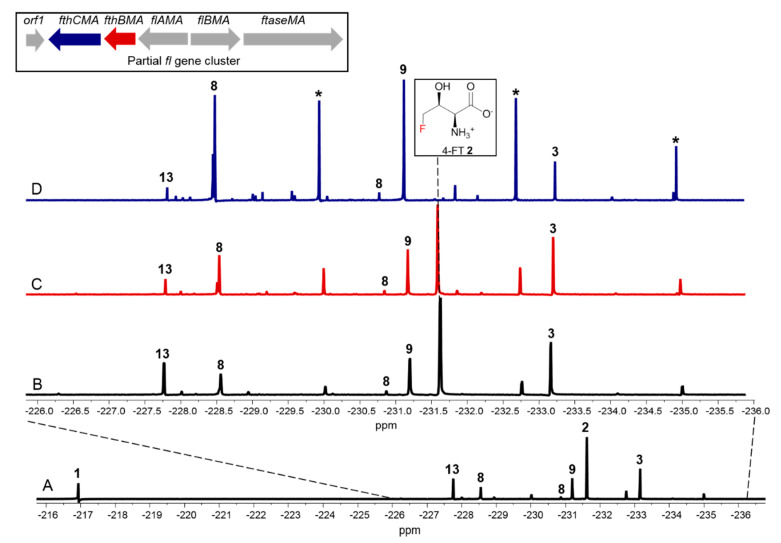
The ^19^F-NMR analysis of fluorometabolites from MA37 wild-type (WT) full fluorometabolite spectrum (**A**), and the comparison of MA37 WT (**B**), MA37_Δ*fthBMA* (**C**) and MA37_Δ*fthCMA* (**D**). The asterisks indicate three unidentified fluorometabolites with increased production in MA37_Δ*fthBMA*.

Considering that MA37 produces a broader spectrum of fluorometabolites compared to *S. cattleya*, the gene homologues of *fthB* and *fthC* in MA37 (*fthBMA* and *fthCMA*, respectively) may have roles in other unidentified fluorometabolites. To this end, we generated two MA37 variants where *fthBMA* and *fthCMA* were subjected to in-frame deletion, respectively. After fermentation (50 mL, 28 °C, 12 d), the supernatants of the culture broths of the two variants along with the wild type (WT) were subjected to ^19^F-NMR analysis. The MA37_Δ*fthBMA* variant demonstrated a relatively similar pattern of secreted **2** as well as other unidentified fluorometabolite production to the WT, consistent with the previous conclusion for *S*. *cattleya* [9] (Figure 2A,B).

Gene inactivation of *fthCMA,* which encodes a putative 4-FT exporter, led to significant reduction of **2** as observed in ^19^F-NMR analysis, also consistent with the previous report [9]. We noticed that **1** together with five other unknown fluorometabolites (compounds with star labels and 5-FDR **8**, **9** (Figure 2D)) showed increased production in the MA37_Δ*fthCMA* variant compared to the ones in the WT (Figure 2A,C and Figure S1). The accumulation of these unidentified fluorometabolites in the MA37_Δ*fthCMA* variant suggested that they are shunt metabolites, arising from aberrant derailment of the main pathways of **1** and **2** in MA37.

Next, we investigated the effect of the biosynthetic genes in the *fdr* BGC on other fluorometabolites, which contain three functionally assigned proteins in the pathway of **3** [2]. FdrA was proposed to be a metal-dependent phosphoesterase that mediates the dephosphorylation of 5-FDRP **11** to generate **8**, the key intermediate in the second biosynthetic pathway leading to the production of **3** [2]. FdrC has been biochemically characterized to be a short-chain dehydrogenase that catalyzes the oxidation of 5-FDR **8**, followed by spontaneous hydrolysis to yield **3**. FdrB is a putative dihydroxyacid dehydratase, an enzyme analogue of SalH in the biosynthesis of salinosporamide A [19], which has been proposed to convert 5-chlororibonate to 5-chloro-4-hydroxy-2-oxopentanoate (Figure S2).

**Figure 3:**
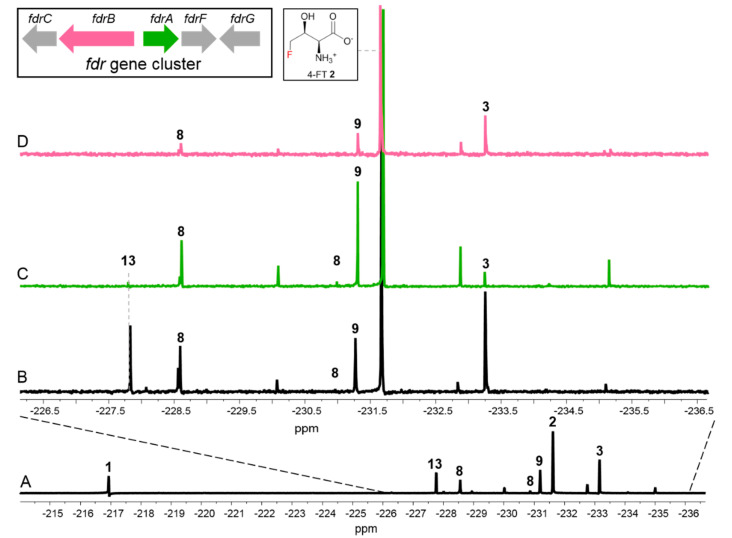
The ^19^F-NMR analysis of fluorometabolites from the MA37 WT full fluorometabolite spectrum (**A**), and the comparison of MA37 WT (**B**), MA37_Δ*fdrA* (**C**) and MA37_Δ*fdrB* (**D**).

To this end, we generated two MA37 variants, Δ*fdrA* and Δ*fdrB*. Comparative fluorometabolite profiling observed in the ^19^F-NMR analysis revealed that the production of **3** in these two variants was reduced compared to in the WT (Figure 3) while **1** and **2** were only mildly affected. Interestingly, the inactivation of individual genes indeed affected other unidentified fluorometabolites. One of the fluorine signals with a significantly different intensity is found at −227.80 ppm (compound **13** in Figure 3C,D). The production of this metabolite was abolished in Δ*fdrA* and Δ*fdrB* variants compared with the one in the WT, suggesting that the production of this metabolite is directly related to the catalytic functions of FdrA and FdrB. One possibility is that this metabolite is 5-fluoro-4-hydroxy-2-oxopentanoate **13**, a dehydrated product generated by the action of FdrB on **3**.

**Figure 4:**
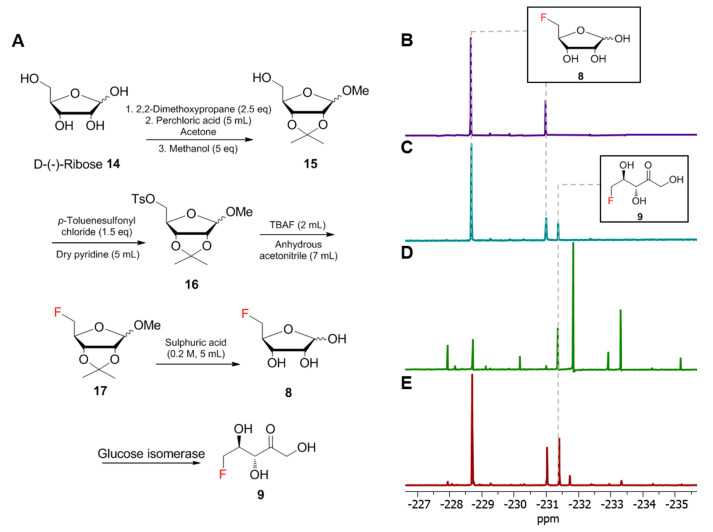
The identification of 5-fluoro-5-deoxy-D-ribulose (5-FDRul) in the fluorometabolite spectrum of MA37. (**A**). The scheme describing the synthesis route using D-(-)-ribose to generate 5-FDRul **9**. (**B**). The synthesis product of 5-FDR. (**C**). The reaction mixture of 5-FDR **8** after incubation with glucose isomerase. (**D**). The aqueous extraction of MA37 wild type fluorometabolites. (**E**). The spiking of (**C**) into (**D**). The synthetic 5-FDRul merged with the peak at −231.42 ppm from MA37 wild-type aqueous extract, confirming it to be one of the fluorometabolites observed from the spectrum.

Another significant fluctuation in the ^19^F-NMR spectra is the signal at −231.42 ppm (compound **9** in Figure 3). While the production level of this metabolite appears to be reduced in the Δ*fdrB* variant compared to the WT (Figure 3A,B), the inactivation of *fdrA* upregulated the production of this metabolite as observed in Figure 3C. Interestingly, this fluorometabolite was also accumulated in the Δ*fthCMA* MA37 variant, suggesting that the synthesis of this fluorometabolite may result from the common intermediates in the biosynthetic pathways of **1**, **2** and **3**. However, the biochemical characterization of the enzymes produced by the genes *fdrA* and *fdrB* was not successful due to production problems in *E. coli* (inclusion bodies). Based on previous studies in *S. cattleya*, the chemical shift of this metabolite in ^19^F-NMR spectrum highly coincided with that of 5-FDRul **9** [17]. Previously, **9** was synthesized to probe the fluorometabolism in *S. cattleya*, and it was found that it is not one of the intermediates or a metabolite in the culture of *S. cattleya*. To confirm whether **9** was indeed a metabolite in MA37, the compound was prepared synthetically according to the protocols in the literature [17]. Moreover, **8** was first chemically prepared from D-ribose according to the literature (Figure 4A and Figures S3–S7) [18], followed by enzymatic conversion to 5-FDRul using commercially available glucose isomerase (Sigma Aldrich UK cat no. G4166). The inspection of ^19^F-NMR indicated that **9** (−231.42 ppm observed in ^19^F-NMR) (Figure 4C and Figures S8 and S9) was indeed generated in a mixture of unreacted α- and β- anomers of **8** (−227.80 and −231.42 ppm, respectively) compared with the synthetic 5-FDR sample (Figure 4B). The ^19^F-NMR signals coincided when the enzyme reaction sample was mixed with the supernatant of MA37 fermentation broth (Figure 4E), confirming that the fluorine signal at −231.42 ppm is **9**. Interestingly, the chemical shifts of both anomers of **8** in the reaction mixture also overlapped with two previously unknown signals in the supernatant of MA37 culture broth as evidenced in the ^19^F-NMR spectra (Figure 4B,E). Taken together, this study identified **9** as one of the unidentified fluorometabolites in MA37, further extending the very small collection of this rare class of bacterial natural products.

**Figure 5:**
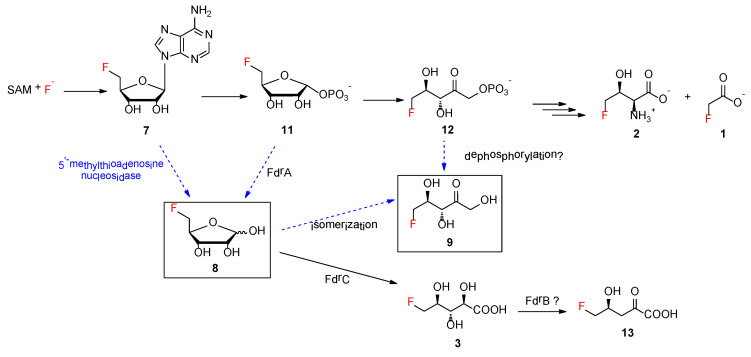
The proposed pathways of identified fluorometabolites in *Streptomyces sp*. MA37. Arrows with solid lines indicate the enzymatic reactions were biochemically confirmed. Arrows with dashed lines indicate biotransformations that remain to be determined. The compounds in boxes are new fluorometabolites identified in this study.

Additionally, **8** was proposed to be a key intermediate in the branched fluorometabolism for generating FHPA as a result of the action of FdrA, a metal-dependent phosphoesterase [2]. The increased accumulation of **8** in the MA37 variants Δ*fdrA* and Δ*fthCMA* suggested that another promiscuous house-keeping enzyme in MA37 is able to generate **8**. One possible enzyme candidate is 5′-methylthioadenosine (MTA) nucleosidase, a key enzyme, in the methionine salvage pathway in microbes, that catalyzes 5′-methylthioadenosine to its corresponding methylthioribose (Figure 5) [20]. Interestingly, it is likely that *S. cattleya* may recruit 5-methylthio-ribose-1-phosphate isomerase, another key enzyme in the methionine salvage pathway, as part of its fluorometabolism toward **1** and **2** (Figure S10) [8]. This indicated that natural product biosynthetic pathways can recruit promiscuous enzymes from primary metabolisms to enable the evolution of biosynthetic capacity and expand the range of known organofluorine biochemistry. A BLAST search using the sequence of MTA nucleosidase from *Streptomyces laurentii* (accession no. BAU88196) as a query against the annotated MA37 draft genome in the RAST server [21] revealed the presence of one gene encoding MTA nucleosidase in MA37 (accession no. MT478135). The encoded protein possesses several crucial amino acids for substrate binding and catalytic function as displayed by multiple sequence alignment. Protein modelling in the Pyre2 server [22] also indicated that the overall predicted structure shares high homologue (100% confidence) with other MTA nucleosidases and purine nucleoside hydrolases (Figure S11). This analysis is in sharp contrast to what has been observed in *S. cattleya* and *S. xinghaiensis.* No such gene encoding MTA nucleosidase can be found in the genomes of either *S. cattleya* or *S. xinghaiensis*. It is known that *S. cattleya* is the producer of **1** and **2** [23] and that *S. xinghaiensis* only produces **1** [23]. In both cases, no other fluorometabolites were found in these bacteria. Our bioinformatics analysis strongly suggests that the putative MTA nucleosidase in MA37 is responsible for the increased accumulation of **8**. MTA nucleosidases catalyze a similar biochemical reaction to that of the purine nucleoside hydrolase identified in the purine salvage pathway that catalyzes adenosine to D-ribose. Previously, a purine nucleoside hydrolase from *Trypanosoma vivax* (TvNH) was shown to mediate the conversion of 5′-FDA to 5-FDR. As a result, it has been utilized as a biocatalyst in one-pot biotransformation involving fluorinase for the synthesis of 5-deoxy-5-[^18^F] fluororibose as a potential diagnostic reagent for positron emission tomography applications [24]. MTA nucleosidase from *E*. *coli* has been shown to be able to convert adenosine to D-ribose, albeit less efficiently than its natural substrate, 5′-methylthioadenosine [25]. Therefore, the putative MTA nucleosidase in MA37 is likely to be responsible for the accumulation of **8** in the MA37 variants. The biochemical characterization of this protein may offer an alternative biocatalyst to the biotransformation of 5-deoxy-5-[^18^F] fluororibose.

The bio-origin of **9** still remains to be biochemically determined. It is worth noting that, although **9** has been synthesized as a chemical probe, **9** is not part of the fluorometabolism in *S. cattleya* [17]. In the case of MA37, **9** is likely to be a shunt product deviating from the two main fluorometabolisms, resulting from the premature isomerization or hydrolysis of reactive intermediates, such as **8** or **12**, respectively (Figure 5). No gene encoding glucose isomerase was found in the draft genome of MA37. Although ribose isomerase, which has been shown to catalyze the conversion of D-ribose to D-ribulose, was purified from extracts of *mycobacterium smegatis* in 1975 [26], its protein sequence remains to be confirmed. One may not exclude the possibility that a homologue of ribose isomerase present in MA37 is responsible for the production of compound **9** in MA37 WT and its variants. Another possibility is that the presence of **9** may result from a promiscuous dephosphorylase enzyme that directly converts **12** to **9**.

## 4. Conclusions

In this study, **9** was found to be the shunt fluorometabolite in MA37 using a combination of genetic inactivation, chemo-enzymatic synthesis and NMR analysis. The inactivation of key biosynthetic genes in both the *fl* and *fdr* gene clusters in MA37 resulted in the divergent production of other unidentified fluorometabolites as observed in ^19^F-NMR analysis. The identification of these two new fluorometabolites in MA37 expands the scope of bacteria-derived fluorometabolites, suggesting the existence of other gene products that are able to metabolize intermediates in the known fluorometabolisms in MA37. Compound **9** is a mono-fluorine-substituted sugar derivative which may serve as an important building block in other chemical and biochemical investigations [27]. Understanding the biochemistry of these metabolites will facilitate the synthesis of fluorinated building blocks through de novo biotransformation from fluoride ions.

## Supplementary Materials

The following are available online at https://www.mdpi.com/2218-273X/10/7/1023/s1, Figure S1: ^19^F-NMR analysis of FAc **1** from MA37 WT (**A**), MA37_Δ*fthBMA* (**B**) and MA37_Δ*fthCMA* (**C**)., Figure S2: The comparison of the fdr pathway (**A**) and the precursor biosynthesis pathway *sal* of salinosporamide A (B), Figure S3: ^1^H-NMR of 1-methoxy-2,2-isopropyliden-α/β-D-ribofuranose **15** (400MHz, CD_3_OD), Figure S4: ^1^H-NMR of 2,3-O-isopropylidene-5-O-(*p*-toluenesulfonyl)-β,D-ribofuranoside **16** (400MHz, CD_3_OD), Figure S5: ^1^H-NMR of 4-fluoromethyl-6-methoxy-2,2-dimethyltetrahydrofuro-1,3-dioxole **17** (400MHz, CD_3_OD), Figure S6: ^19^F-NMR of 5-deoxy-5-fluoro-2,3-O-isopropylidene-β-D-ribofuranosid **17** (400MHz, D_2_O), Figure S7: ^19^F-NMR of 5-FDR **8** (400MHz, D_2_O), Figure S8: Decoupling ^19^F-NMR of generation of 5-FDRul **9** (400MHz, D_2_O), Figure S9: The coupling ^19^F-NMR spectrum of 5-FDR **8**, Figure S10: **A**. The biochemical reaction of MTA nucleosidase in primary metabolism. **B**. The putative recruitment of 5-methylthio-ribose-1-phosphate isomerase from methionine salvage pathway as part of *Streptomyces cattleya*’s fluorometabolism. **C**. The proposed incorporation of MTA nucleosidase in **3** biosynthesis in MA37, Figure S11, The comparison of the predicted structure of MTA nucleosidase from MA37 (**A**) with the one from *Campylobacter jejuni* (**B**) (PDB No. 6AYT), suggesting the high degree of similarity of their overall structures and the highly conserved catalytic dyad (Ser 162-Asp-163 in the case of MTA nucleosidase from MA37) and the substrate binding pocket (Met139-Glu140). **C**. The multiple amino acid sequences alignment of the MTA nucleosidase found in MA37 with several other well-characterized MTA nucleosidase, showing the conserved catalytic dyad (Ser 162-Asp-163) and substrate binding pocket (Met139-Glu140).
