# Supplementary material for: Identification of 5-Fluoro-5-Deoxy-Ribulose as a Shunt Fluorometabolite in Streptomyces sp. MA37
Source: Biomolecules. 2020 Jul 10;10(7):1023. doi: 10.3390/biom10071023 (PMC7408626; doi:10.3390/biom10071023)
Supplement: Supplementary file 1 [file biomolecules-10-01023-s001.pdf]

Identification of 5-Fluoro-5-Deoxy-Ribulose as a Shunt Fluorometabolite in *Streptomyces sp*. MA37

Linrui Wu [1], Ming Him Tong [1], Kwaku Kyeremeh [2] and Hai Deng [1,*]

1 Department of Chemistry, University of Aberdeen, Aberdeen AB24 3UE, UK; linrui.wu3@abdn.ac.uk (L.W.); r01mht13@abdn.ac.uk (M.H.T.)
2 Department of Chemistry, University of Ghana, P.O. Box LG56 Legon-Accra, Ghana; kkyeremeh@ug.edu.gh
* Correspondence: h.deng@abdn.ac.uk

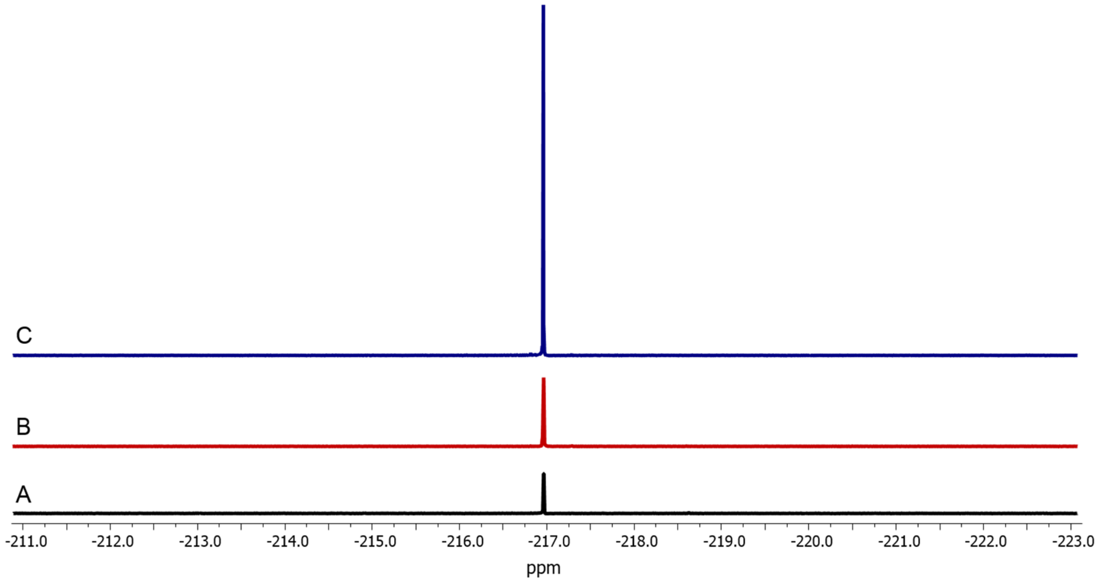

Figure S1. ^{19}F-NMR analysis of FAc **1** from MA37 WT (A), MA37_Δ*fthBMA* (B) and MA37_Δ*fthCMA* (C).

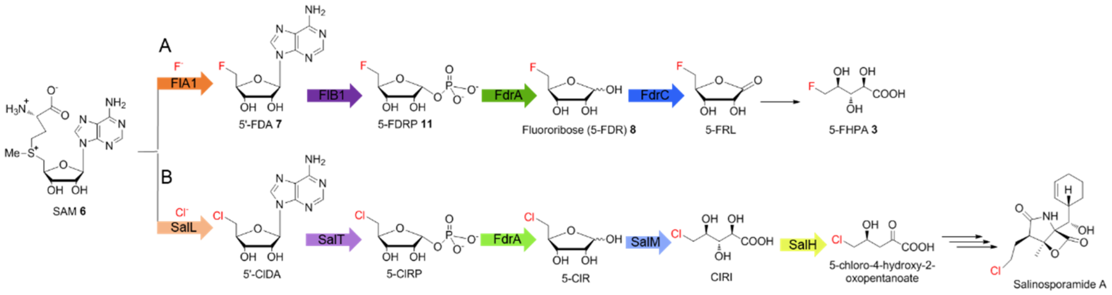

Figure S2. The comparison of the *fdr* pathway (**A**) and the precursor biosynthesis pathway *sal* of salinosporamide A (**B**) [1].

The synthesis of 1-methoxy-2,2-isopropyliden-α/β-D-ribofuranose **15**

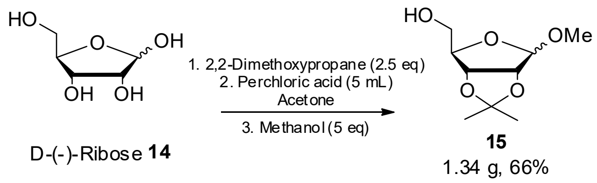

^1H NMR (400 MHz, $CDCl_3$) δ 4.93 (s, 1 H, $C_{1'}H$), 4.78 (d, J=6.0 Hz, 1H, $C_{2'}H$), 4.56 (d, J=6.0 Hz, 1H, $C_{3'}H$), 4.39-4.34 (m, 1H, $C_{4'}H$), 3.68-3.53 (m, 2H, $C_{5'}H_2$), 3.39 (s, 3H, OCH_3), 1.45 (s, 3H, CH_3), 1.28 (s, 3H, CH_3). Comparison of 1H-NMR spectral data with the one reported (ref) demonstrated that the synthetic compound is indeed the target.

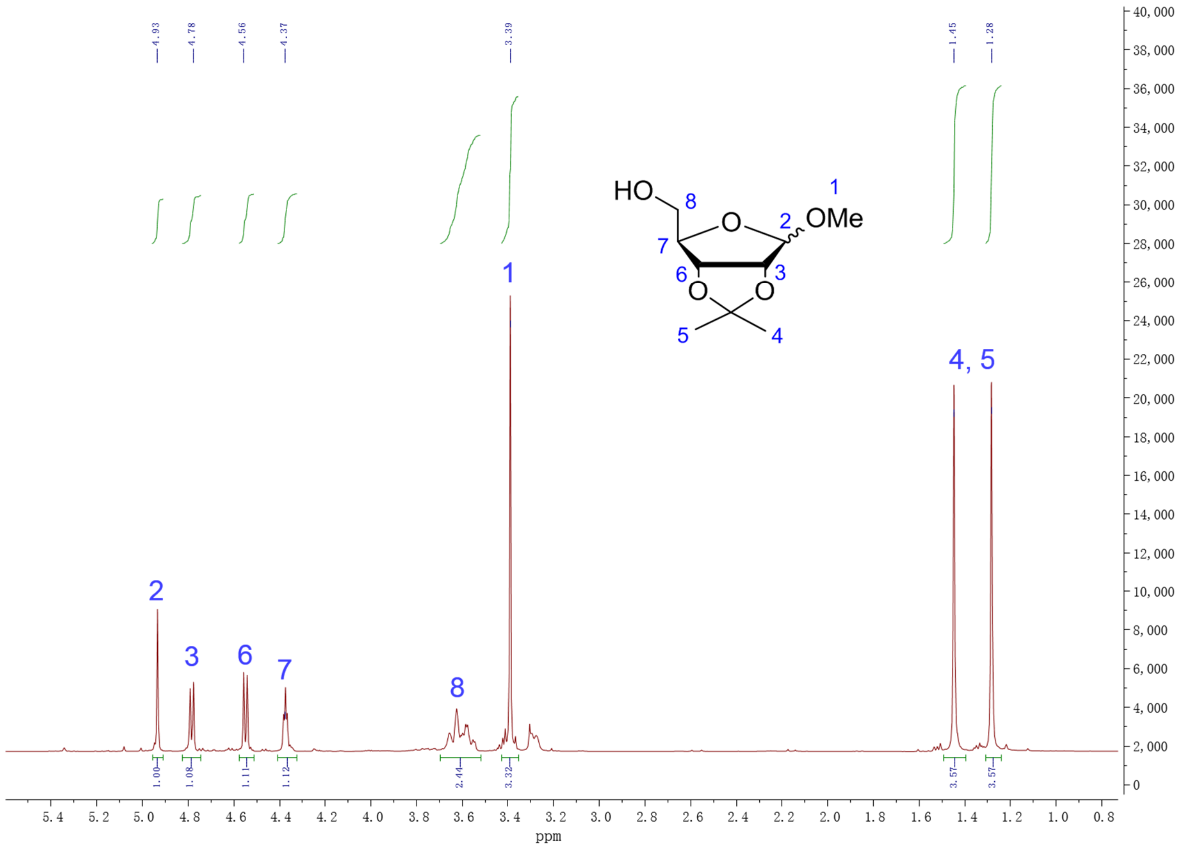

Figure S3. ^1H-NMR of 1-methoxy-2,2-isopropyliden-α/β-D-ribofuranose **15** (400MHz, CD_3OD).

The synthesis of methyl 2,3-O-isopropylidene-5-O-(*p*-toluenesulfonyl)-β,D-ribofuranoside **16**

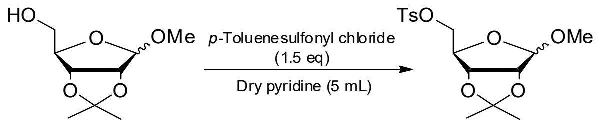

15 **16**
1.21g, 69%

^{1}H NMR (400 MHz, $CDCl_3$) δ 7.82 (d, J=8.0 Hz, 3.0 Hz, 2H, aromatic), 7.38 (d, J=8.0 Hz, 2H, aromatic), 4.94 (s, 1H, H-1), 4.61 (d, J=5.9, 1H), 4.55 (d, J=5.9, 1H, H-2), 4.32 (t, J=7.5 Hz, 1H, H-4), 4.01 (m, 2H, H-5), 3.24 (s, 3H, OCH_3), 2.46 (s, 3H, Ar-CH_3), 1.45 (s, 3H, -CCH_3), 1.29 (s, 3H, -CCH_3). Comparison of 1H-NMR spectral data with the one reported (ref) demonstrated that the synthetic compound is indeed the target.

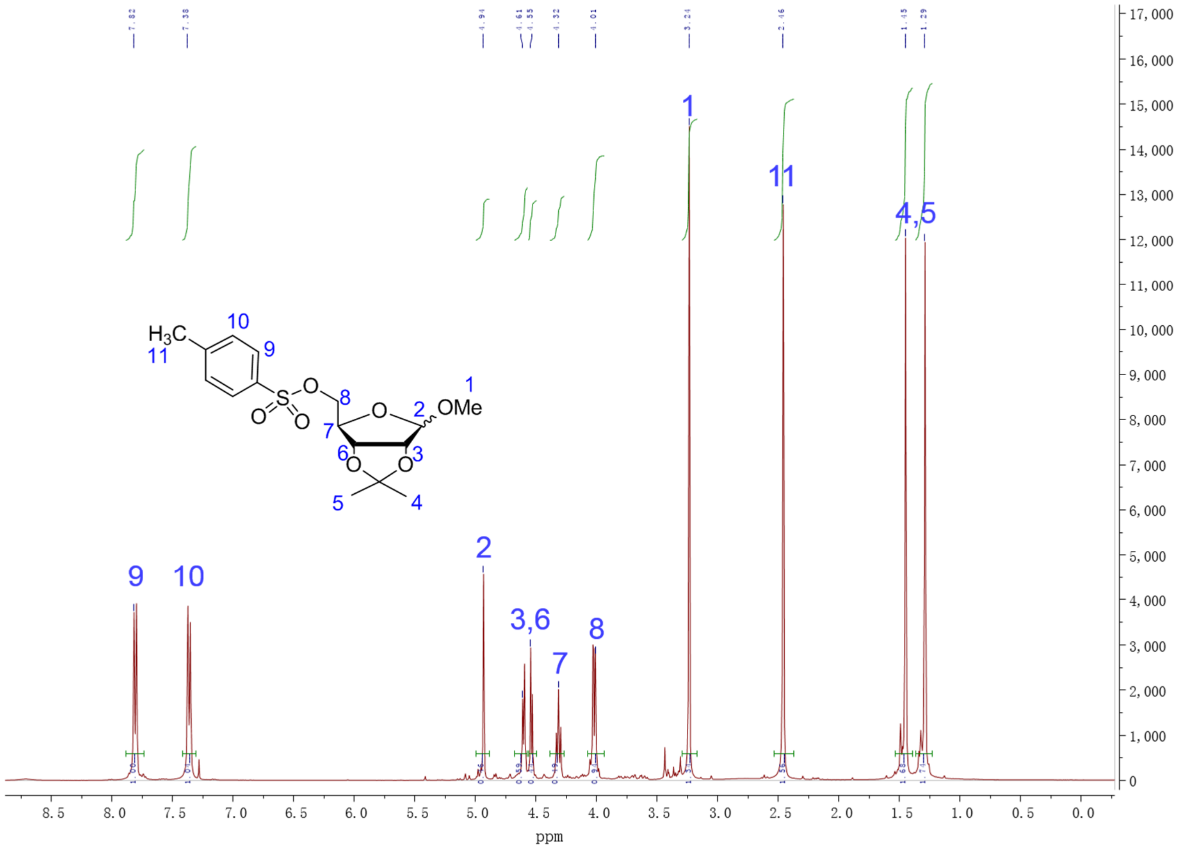

Figure S4. ^{1}H-NMR of 2,3-O-isopropylidene-5-O-(*p*-toluenesulfonyl)-β,D-ribofuranoside **16** (400MHz, CD_3OD).

The synthesis of 5-deoxy-5-fluoro-2,3-O-isopropylidene-β-D-ribofuranosid **17**

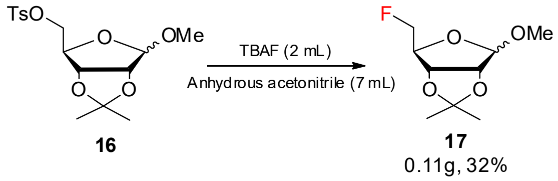

^{1}H NMR (400 MHz, $CDCl_3$) δ 4.91 (d, 1H, J=3.0 Hz, H-1), 4.62 (d, 1H, J=3.0 Hz, H-2), 4.51 (d, 1H, J=3.0 Hz, H-3), 4.20-4.18 (m, 2H, H-4 H-5), 3.25 (s, 3H, OCH_3), 1.41 (s, 3H, $-CCH_3$), 1.25 (s, 3H, $-CCH_3$).

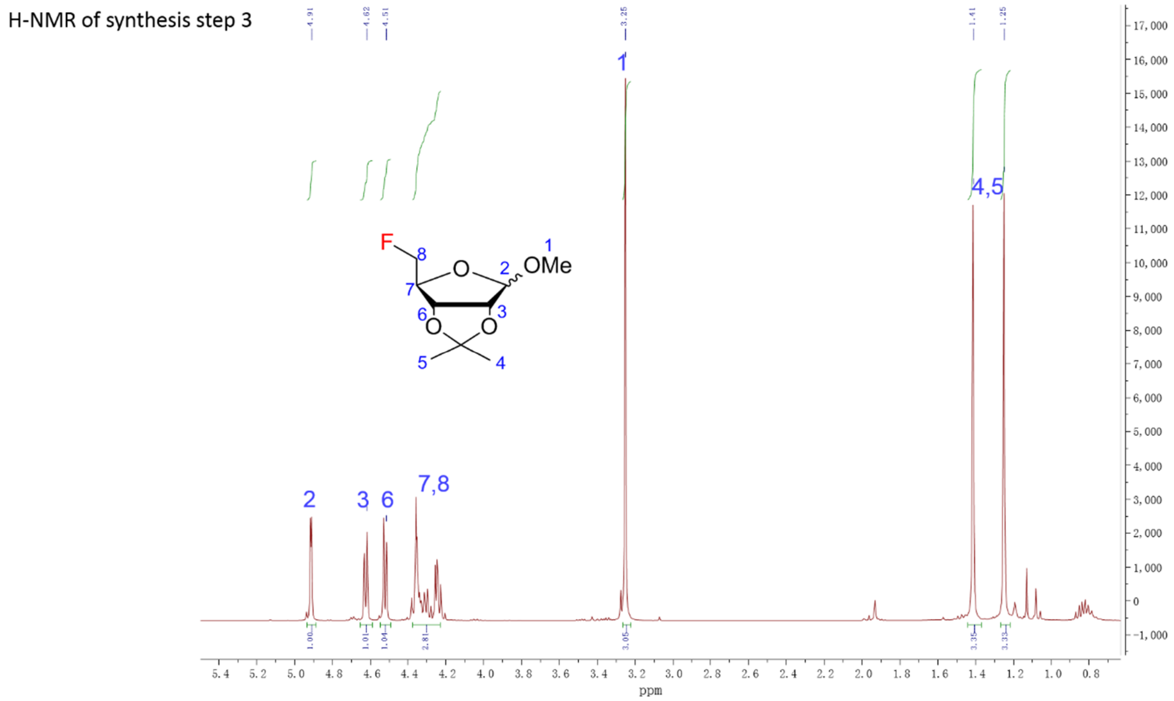

Figure S5. ^{1}H-NMR of 4-fluoromethyl-6-methoxy-2,2-dimethyltetrahydrofuro-1,3-dioxole **17** (400MHz, CD_3OD).

^{19}F-NMR (400 MHz, D_2O) δ -225.03

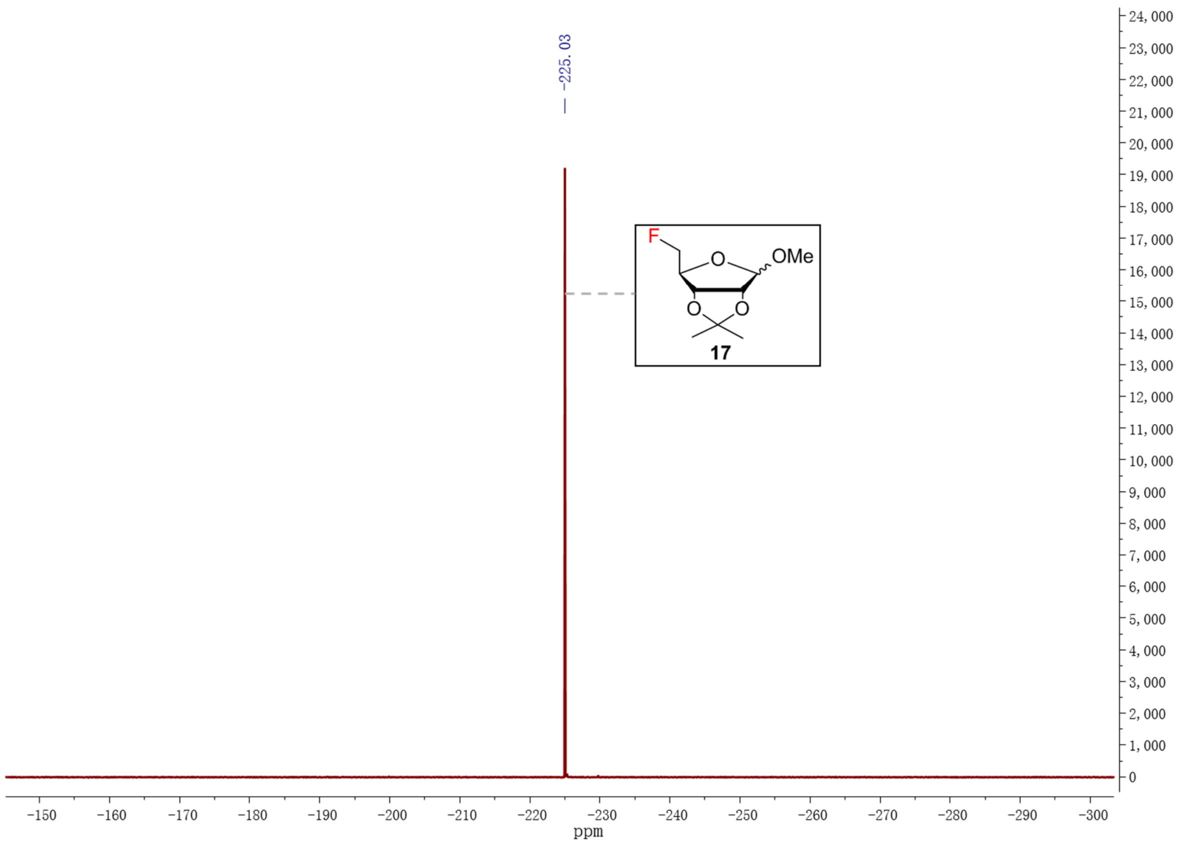

Figure S6. ^{19}F-NMR of 5-deoxy-5-fluoro-2,3-O-isopropylidene-β-D-ribofuranosid **17** (400MHz, D_2O).

The synthesis of 5-FDR **8**

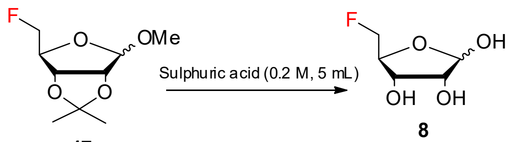

^{19}F-NMR (400 MHz, D₂O) δ -228.72, -231.04

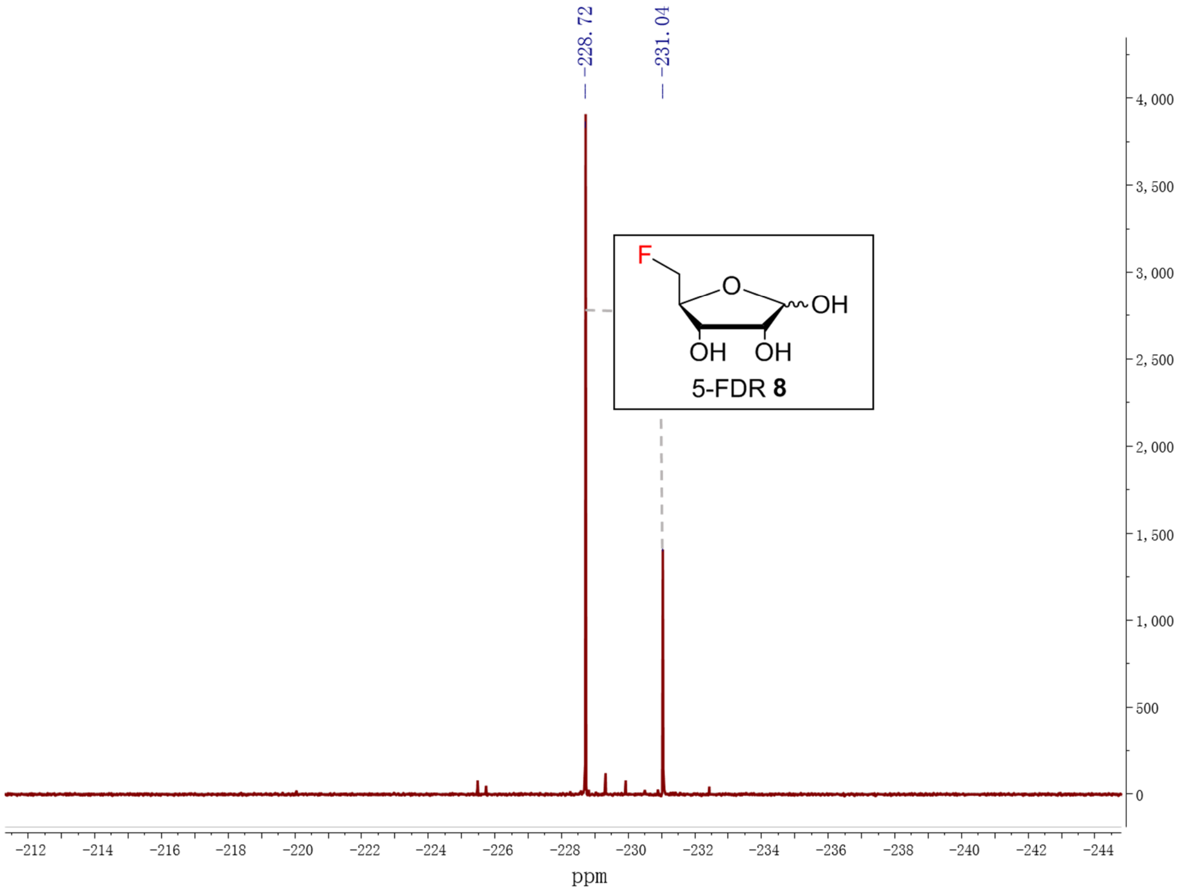

Figure S7. ^{19}F-NMR of 5-FDR **8** (400MHz, D_2O).

The generation of 5-FDRul **9**
^{19}F-NMR (400 MHz, D_2O) δ -231.42.

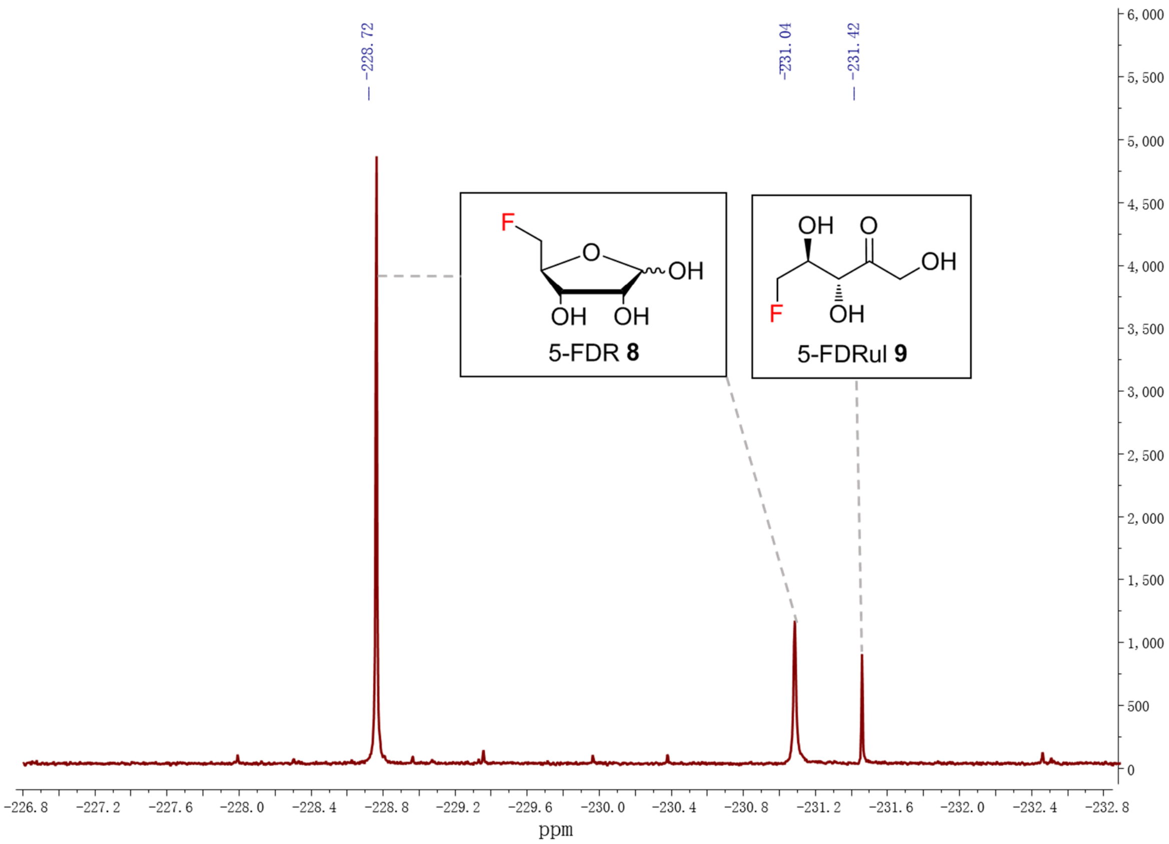

Figure S8. Decoupling ^{19}F-NMR of generation of 5-FDRul **9** (400MHz, D_2O).

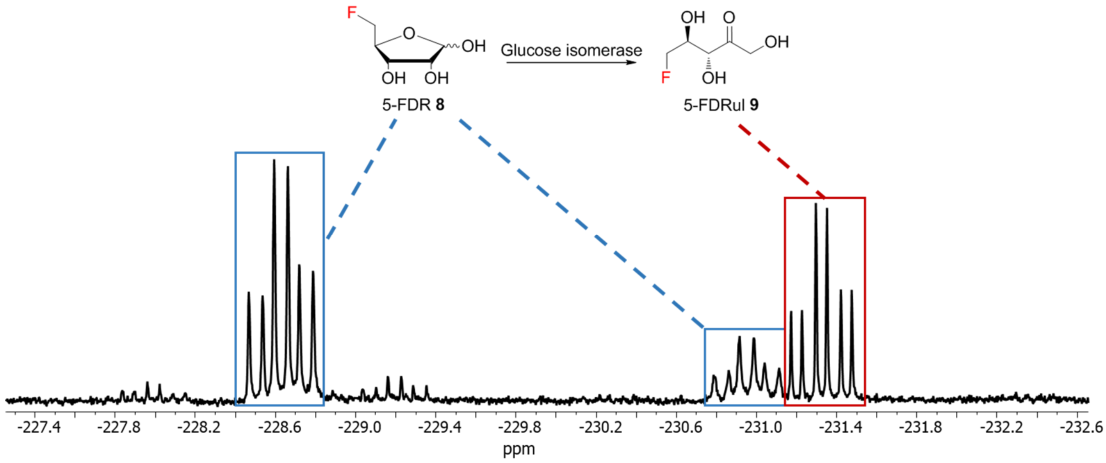

Figure S9. The coupling ^{19}F-NMR spectrum of 5-FDR **8** (*dt*, δ_F-228.63, $^2J_{HF}$ 28.0 Hz, $^3J_{HF}$ 48.0 Hz, *dt*, δ_F-230.95, $^2J_{HF}$ 28.0 Hz, $^3J_{HF}$ 48.0 Hz) after 6 h incubation with glucose isomerase, showing the accumulation of 5-FDRul **9** (*dt*, δ_F-231.33, $^2J_{HF}$ 24.0 Hz, $^3J_{HF}$ 48.0 Hz).

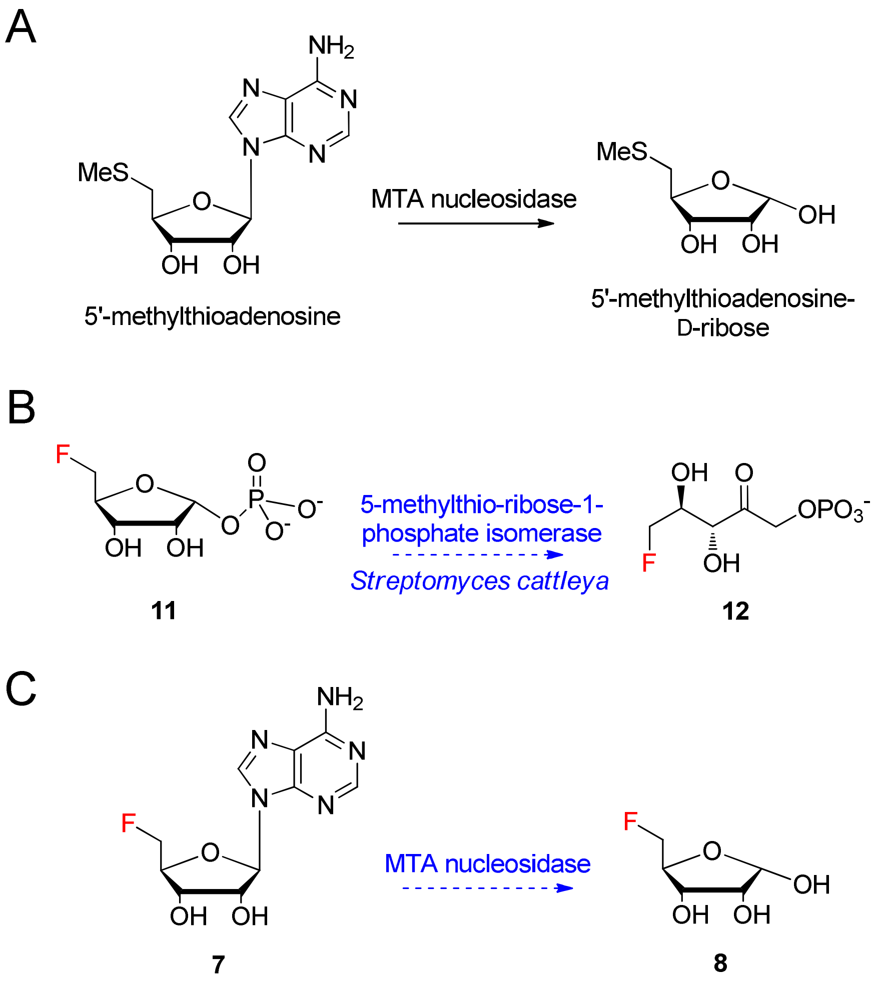

Figure S10. A. The biochemical reaction of MTA nucleosidase in primary metabolism. **B**. The proposed recruitment of 5-methylthio-ribose-1-phosphate isomerase from methionine salvage pathway as part of *Streptomyces cattleya*'s fluorometabolism. **C**. The propo[sed incorporation of MTA nucleosidase in **3** biosynthesis in MA37.

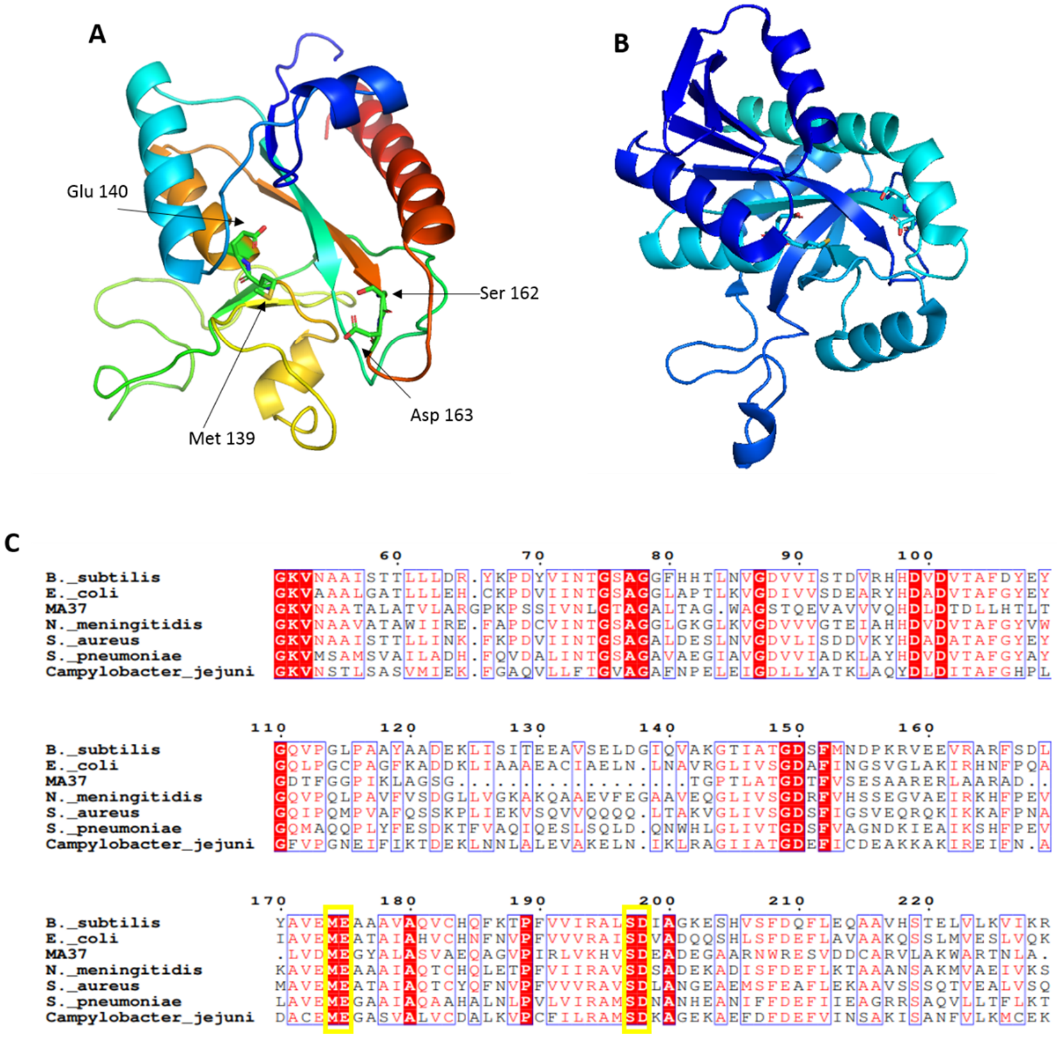

Figure S11. The comparison of the predicted structure of MTA nucleosidase from MA37 (**A**) with the one from *Campylobacter jejuni* (**B**) (PDB No. 6AYT) [2], suggesting the high degree of similarity of their overall structures and the highly conserved catalytic dyad (Ser 162-Asp-163 in the case of MTA nucleosidase from MA37) and the substrate binding pocket (Met139-Glu140). **C**. The multiple amino acid sequences alignment of the MTA nucleosidase found in MA37 with several other well-characterized MTA nucleosidase, showing the conserved catalytic dyad (Ser 162-Asp-163) and substrate binding pocket (Met139-Glu140).

Reference

1. Eustáquio, A.S.; McGlinchey, R.P.; Liu, Y.; Hazzard, C.; Beer, L.L.; Florova, G.; Alhamadsheh, M.M.; Lechner, A.; Kale, A.J.; Kobayashi, Y. Biosynthesis of the salinosporamide A polyketide synthase substrate chloroethylmalonyl-coenzyme A from *S*-adenosyl-L-methionine. *Proc. Natl. Acad. Sci. U. S. A.* **2009**, *106*, 12295-12300.
2. Ducati, R.G.; Harijan, R.K.; Cameron, S.A.; Tyler, P.C.; Evans, G.B.; Schramm, V.L. Transition-state analogues of *Campylobacter jejuni* 5'-methylthioadenosine nucleosidase. *ACS chemical biology* **2018**, *13*, 3173-3183.
